# Queryable Gaseous Adsorption Properties of Pure Components and Mixtures in Metal–Organic Frameworks

**DOI:** 10.1021/acs.langmuir.5c03717

**Published:** 2025-11-22

**Authors:** Jui Tu, Chi-Chun Tang, Pi-Chien Chuang, Li-Chiang Lin

**Affiliations:** † Department of Chemical Engineering, 33561 National Taiwan University, Taipei 106319, Taiwan; ‡ William G. Lowrie Department of Chemical and Biomolecular Engineering, the Ohio State University, Columbus, Ohio 43210, United States

## Abstract

Grand canonical Monte Carlo (GCMC) has been the most common simulation method for high-throughput screening of metal–organic frameworks (MOFs) in adsorption applications. However, GCMC results are inevitably limited to specific pressure and temperature conditions, limiting their applicability across the varying conditions in industrial operations. To address this, we develop a macrostate-probability-distribution-based (MPD-based) adsorption database for several key gas molecules (i.e., CO_2_, N_2_, CH_4_, and CO) in approximately 2900 rigid MOF structures. Crucially, MPD computed from flat histogram Monte Carlo can be analytically reweighted to any pressures and temperatures, enabling instant access to adsorption uptakes under any desired conditions. By integrating the ideal adsorbed solution theory (IAST), the database also provides mixture isotherms at user-defined compositions to allow efficient assessment of multicomponent separations. With the MPD-based database, we demonstrate its capability in rapid screening and uncovering structure-performance relationships that govern separation behavior under different operational conditions. Overall, by employing MPD, we present a versatile and interactive adsorption database to facilitate the future discovery of optimal MOFs.

## Graphical abstract

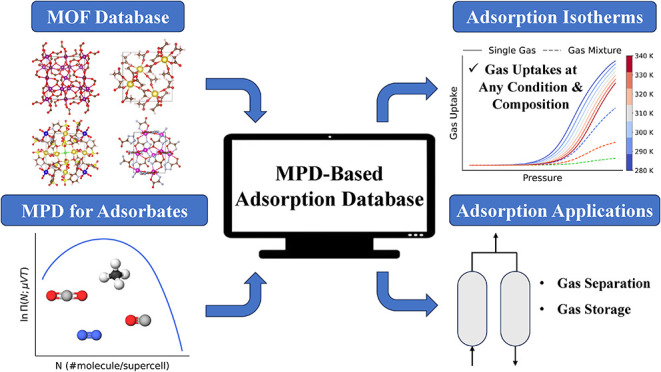

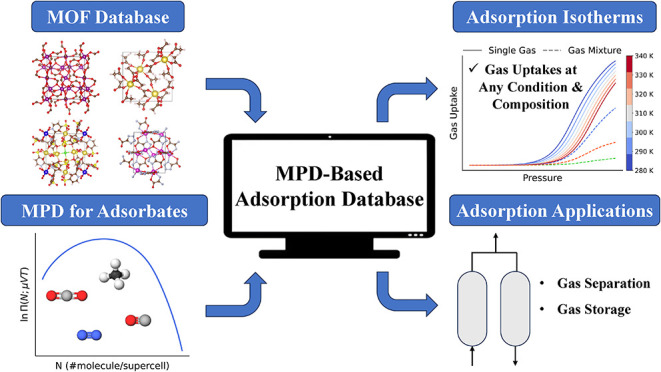

## Introduction

Adsorption technology has become a cornerstone of modern industrial processes, essential for applications such as methane storage, carbon dioxide capture, and natural gas purification, with wide-reaching implications for environmental protection and energy efficiency.^1–8^ Over the past decades, seeking for more efficient adsorption-related processes, particularly the development of novel materials, has been the focus in both academia and industry. Nanoporous materials have drawn considerable interest as promising adsorbents owing to their large surface area and selective adsorption capabilities.^9–11^ Specifically, metal–organic frameworks (MOFs), composed of metal clusters and organic linkers, have gained particular attention for their highly tunable nature.^12–17^ Through manipulating the combination of metal clusters and organic linkers, the pore structures and chemical characteristics of MOFs could be tuned for enhanced adsorption and separation performance. Since the first MOF was synthesized three decades ago,^18^ tens of thousands of MOFs have been reported^19^ and orders of magnitude more could be potentially achieved.^20,21^ To date, numerous structural databases have also been compiled and made publicly available, which include, but are not limited to, computational-ready experimental (CoRE) MOFs,^22–24^ quantum MOFs (QMOFs),^25^ hypothetical MOFs (hMOFs),^26^ Cambridge Structural Database (CSD) MOFs,^27^ and Topologically Based Crystal Constructor (ToBaCCo) MOFs.^28^ They include a wealth of MOFs that have been experimentally reported or can be potentially synthesized. Leveraging these databases, computational high-throughput screening (HTS) utilizing state-of-the-art molecular simulations has been extensively conducted to aid the identification of optimal MOFs for specific adsorption-related applications,^29–37^ significantly reducing the needed resources and time in their developments.

In most, if not all, of the reported HTS studies, Monte Carlo simulations in the grand canonical ensemble (GCMC) have been nearly exclusively adopted to calculate gas adsorption properties.^38–40^ As examples, Wilmer et al. conducted GCMC simulations to study over 130,000 structures from the hMOF database for both methane storage^26^ and natural gas (CO_2_/CH_4_) separation.^41^ For methane storage, hundreds of top MOFs were identified, with methyl-functionalized structures exhibiting enhanced performance. In the context of CO_2_ separation from natural gas (typically composed of 30–90% CH_4_), they computed working capacities, regenerability, and selectivities under targeted conditions. The analysis revealed that optimal MOFs for separation tend to feature narrow pores (4 Å < PLD < 8 Å) and moderate surface areas (1000–2500 m^2^/g). Similarly, for flue gas separation, primarily comprising CO_2_ (8–15%) and inert N_2_ (70–90%), Boyd et al. also assessed flue gas selectivities in 325,000 hMOFs by GCMC simulations under vacuum temperature swing adsorption (VTSA) conditions, identifying 8325 promising structures for further investigations.^42^ Their findings highlighted distinct pore shapes and binding site chemistries that result in notably low Henry’s constants for H_2_O. As for CO/CO_2_ separation, Sung and Lin also performed GCMC simulations on approximately 6000 MOFs in the CoRE MOF database to compute their mixture uptakes and selectivities.^43^ Over 700 MOFs were identified to offer a CO_2_-to-CO selectivity larger than 100. Moreover, correlation between structural features and adsorption properties was also examined and established.

To facilitate future discoveries, several seminal HTS studies have also released their computed adsorption data, allowing peer researchers to further mine the data for more insights or to identify optimal MOFs for related applications. As an example, Keskin and co-workers constructed an online database offering the selectivity, adsorbent performance score (APS), and regenerability of 3816 MOFs within the CSD for separating CO_2_/N_2_ (15/85) mixture.^44^ Additionally, Keskin’s group also performed GCMC simulations and reported the adsorption uptakes of CH_4_ and H_2_ for those MOFs, among the 3490 common MOFs in both the CoRE and CSD databases, that exhibit different gas uptakes depending on the database source.^45^ Besides, Snurr and co-workers also established a large-scale online database that consisted of 160,000 MOFs and 286 zeolites.^46^ Over three million adsorption data points for various gas molecules are included in the database. Additionally, Oliveira et al. explored the influence of force field parameters and partial charges on adsorption properties of 690 MOFs from the CoRE MOF 2014 database for CO_2_ and N_2_.^47^ GCMC simulations were conducted with two force fields, six partial charge schemes at three different temperatures, and the obtained results have also been made publicly available.

While significant progress has been made thus far, a notable limitation of those large databases is their condition-dependent nature. That is, adsorption properties that have been made available are under specific set of conditions (i.e., pressure and temperature). Although one may employ certain adsorption models, such as the Langmuir isotherm,^48,49^ to extrapolate gas uptake to different pressures, limitations still exist, particularly in scenarios where the adsorption isotherms may not be adequately described by the adopted models or when temperature extrapolation is infeasible. For mixtures, even for the same pair to be separated, it is common that the composition and condition of the mixture may differ per geographic locations. For instance, the CO_2_ content in natural gas varies worldwide, ranging from as small as 4% to as large as 50%.^50^ Besides, to fully harness the potential of a material, process-level optimization must be conducted. This requires detailed knowledge of its adsorption properties under varied operating conditions and may involve considerations of different temperatures.^49,51^ As a result, researchers sometimes simply opt for recomputing properties per their targeted condition.

The above-noted limitation arises from the fact that GCMC directly yields the average uptakes. For the adsorption of a pure component, the average uptake ⟨*N*⟩ under a given condition (i.e., pressure and temperature; essentially given chemical potential μ and temperature *T* with a rigid framework structure of a volume *V*) is the result of the probability-weighted average of each possible macrostate (i.e., *N*, the number of molecules in the adsorbent) as shown in eq 1.

$${\left\langle N\right\rangle }_{\mu VT}=\frac{\overset{\infty }{\underset{N=0}{\sum }}N\cdot \Pi \left(N;\mu VT\right)}{\overset{\infty }{\underset{N=0}{\sum }}\Pi \left(N;\mu VT\right)}$$ 1

In this equation, Π­(*N*; μ*VT*) represents the probability of finding the macrostate *N* under the given condition. As noted above, GCMC simulations directly yield the average uptake, which is condition dependent and may not be easily extrapolated to different conditions. By contrast, Π­(*N*; μ*VT*), the so-called macrostate probability distribution (MPD) that can be computed from flat histogram Monte Carlo approaches, can be analytically reweighted per statistical thermodynamics to any pressures (i.e., essentially chemical potentials) and temperatures.^52–56^ That is, by obtaining the MPD, the above-mentioned limitation can be avoided. In other words, an MPD-based adsorption database can be exceptionally versatile, offering adsorption properties at any desired conditions.

To this end, this study for the first time constructs an MPD-based large-scale adsorption database. This MPD-based database includes the pure-component adsorption isotherms across a wide temperature range for four energy- and environment-related adsorbates (i.e., CO_2_, N_2_, CH_4_, and CO) in ∼2900 MOFs from the so-called CoRE MOF 2014 DDEC Database by Nazarian et al.^57^ These MOFs are selected from the widely studied CoRE MOF 2014 database^23^ with high-fidelity atomic charges assigned using the DDEC method.^58^ Their structures are assumed to be rigid. While this rigid assumption may not hold for every single MOF, it is computationally prohibitive to consider this aspect in the development of a large adsorption database as reported herein. Moreover, the ideal adsorbed solution theory (IAST)^59^ is further incorporated herein to predict binary and ternary mixture isotherms at any desired conditions. While MPD can be extended to multicomponent systems as will be discussed later, the added cost is impractical at database scale. With the most versatile database to date at our disposal, a case study to explore the potential of MOFs for flue gas separation is conducted to showcase its capability. Along with this study, an interactive Web site for public access to this database is also made available.

## Computational Details

In this study, a gaseous adsorption database for CO_2_, N_2_, CH_4_, and CO in approximately 2900 MOF structures is established as a proof of concept. The MOFs are selected as noted above from the 2014 CoRE MOF database.^23^ Moreover, high quality point charges, leveraging plane-wave DFT^60^ calculations with the DDEC charge partitioning method,^58^ have also been made available by Nazarian et al.^57^ for accurate descriptions in their electrostatic potential. This makes the MOF set particularly well-suited for adsorption simulations. The structural properties such as pore limiting diameter (PLD), the largest cavity diameter (LCD), accessible surface area (ASA) of MOFs included herein are also determined via the Zeo++ package;^61^ they will be used in the case study presented in this article to establish structure-property relationships.

To compute MPD for a certain adsorbate in each MOF, flat histogram Monte Carlo approaches are employed. Specifically, one of the variants—the so-called NVT + W first reported by Smit and co-workers^55,56^ —is adopted. This method samples each possible macrostate *N* (i.e., *N* molecules in the simulation supercell with a fixed volume *V*) equally under a reference chemical potential (μ) and temperature (*T*). Herein, *N* ranges from 0 (empty) to *N*_max_ (fully saturated)_._ Specifically, for each macrostate *N*, a separate canonical simulation (fixed *N*, *V*, *T*) is conducted. During each NVT + W simulation, Widom ghost insertion or deletion moves are carried out on the fly, but are never accepted. At the same time, the acceptance ratios of each Widom ghost move per grand canonical ensemble are accumulated in the C-matrix during these calculations as shown in eqs 2 and 3, respectively. In this sense, the procedure can be viewed as a quasi-parametrization over *N*, with one independent *NVT* + W simulation per macrostate.

$$\begin{array}{c} C(N\to N+1)\leftarrow C(N\to N+1)+acc\left(N\to N+1\right)\\ C(N\to N)\leftarrow C(N\to N)+(1-acc\left(N\to N+1\right) \end{array}$$ 2

$$\begin{array}{c} C(N\to N-1)\leftarrow C\left(N\to N-1\right)+acc\left(N\to N-1\right)\\ C(N\to N)\leftarrow C\left(N\to N\right)+\left(1-acc\left(N\to N-1\right)\right) \end{array}$$ 3

Herein, the symbol, ←, shown in eqs 2 and 3 above represent the update of the C-matrix element after a ghost insertion and deletion attempt, respectively. For each macrostate, the C-matrix is a tridiagonal matrix with elements of *C*(*N* → *N* + 1),*C*(*N* → *N*), and *C*(*N* → *N* – 1). Subsequently, the transition probability can be computed per eq 4 shown below

4 $$P\left(N\to N_{p}\right)=\frac{C\left(N\to N_{p}\right)}{\sum_{\Delta \in \left\{1,0,-1\right\}}C\left(N\to N+\Delta \right)}$$

Here, *P*(*N* → *N*_p_) refers to the transition probability from one macrostate (*N*) to another proximal macrostate (*N*_p_) (i.e., from *N* to *N* + 1 or *N* – 1, respectively). Finally, with the detailed balance as shown in eq 5, the MPD or Π­(*N*; μ*VT*), which represents the probability to identify the macrostate *N*, can be computed.

5 $$P\left(N\to N_{p}\right)\Pi \left(N;\mu ,V,T\right)=P\left(N_{p}\to N\right)\Pi \left(N_{p};\mu ,V,T\right)$$

With the MPD under a given reference condition (i.e., Π­(*N*; μ*VT*)), it can be further reweighted analytically as shown in eq 6 to different fugacity (*f* ^′^) and temperature (i.e., *T* ^′^). Essentially, the MPD under any other conditions (i.e., μ^′^,*V*,*T* ^′^) can be obtained.

6 $$\ln\frac{\Pi \left(N;\mu^{\prime },V,T^{\prime }\right)}{\Pi \left(0;\mu^{\prime },V,T^{\prime }\right)}=\ln\frac{\Pi \left(N;\mu ,V,T\right)}{\Pi \left(0;\mu ,V,T\right)}+N\cdot \ln\frac{f^{\prime }}{f}+N\cdot \ln\frac{\beta^{\prime }}{\beta }+\sum_{n=1}^{\infty }\frac{1}{n!}\frac{\partial^{n}\ln Q_{c}}{\partial \beta^{n}}{\left(\beta^{\prime }-\beta \right)}^{n}$$

In eq 6, *Q*_c_ denotes the configurational part of the canonical partition function and β equals to 1/*k*_B_ *T*. Additionally, eq 6 involves a Taylor expansion in temperature about the reference *T*, where *n* orders of the expansion terms can be included, while the dependence on pressure (i.e., chemical potential) is treated exactly. Herein, only the first-order term is included as prior study showed that using the first-order term alone is sufficiently accurate over the temperature range considered in this study.^62^ As will be shown below, tests conducted in this study also validate this approximation. More detailed derivations can be found somewhere else in the literature.^55^

*NVT* + W simulations with a reference temperature and fugacity of 300 K and 0.1 bar, respectively, are conducted using the RASPA code^63^ with in house modifications. The maximum molecular number *N*_max_ of an adsorbate of interest in each studied framework is set to be the corresponding saturation loading *N*_sat_ determined by GCMC simulations under a large fugacity of 100 bar and at a low temperature 275 K. This choice offers a high driving force to achieve a saturated state, ensuring a sufficiently large *N*_max_ value. GCMC simulations are also conducted with results serving as benchmark references. Convergence of the *NVT* + W sampling is set by accuracy against GCMC in a set of selected structures spanning wide Henry’s constants. We choose the minimal number of *NVT* + W cycles that consistently keeps MPD-based uptakes within 5% of GCMC-computed ones. In these calculations, both adsorbate–adsorbate and adsorbate-MOF interactions are described with the Lennard-Jones (LJ) potential and Coulombic interactions. The LJ parameters for MOF atoms are adopted from the universal force field (UFF).^64^ This generic, nonpolarizable force field has been widely used for MOFs and has shown good agreement with experimental uptakes in prior studies.^39^ Although some polarizable force fields may offer improved accuracy in select cases,^65,66^ their computational cost is unsuitable for the present database scale. Atomic charges are assigned using the reported DDEC charges associated with the CoRE MOF 2014 DDEC database.^57^ For gas molecules, the TraPPE^67,68^ models are used to describe CO_2_, N_2_, and CH_4_ for their LJ parameters and partial charges, while the ESP-MM^69^ developed by Cho et al. is adopted for CO. Details of their parameters can be found in the Supporting Information. The LJ potential is shifted at a set cutoff radius, while Columbic interactions are computed via Ewald summation with an accuracy of 10^–6^. Regarding the adopted cutoff distance, a value of 12.8 Å without tail corrections is used as the default setting. However, for MOFs with a unit cell dimension to be between 10.0 Å and 12.8 Å, we instead apply a 10.0 Å cutoff distance with tail corrections in order to reduce the needed number of unit cells and thus lower the computational cost. A previous study by Smit and co-workers showed that, when tail correction is employed, the computed adsorption properties are not a strong function of the adopted cutoff value.^70^ This overall offers reliable accuracy while maintaining computational efficiency. As an example, for MOF LEWVAL with a perpendicular unit cell dimension of 8.8 × 11.5 × 11.7 Å^3^, a large supercell comprising 3 × 3 × 3 unit cells is required if a cutoff distance of 12.8 Å is used. With a reduced cutoff distance of 10.0 Å, a smaller supercell of 3 × 2 × 2 unit cells is instead needed, leading to at least 7-fold lower computational cost. As shown in Figure S1, both cutoff settings yield highly consistent results, validating the accuracy and efficiency of this adopted approach.

## Results and Discussion

This section first introduces the sampling and interpolation approach adopted in this study to develop a large-scale MPD-based adsorption database, followed by its extensive validation. The incorporation of the ideal adsorbed solution theory (IAST) is subsequently detailed to predict binary and even ternary mixtures. An interactive online platform is then introduced, allowing users to explore structural properties and download pure and mixture isotherms for over 2900 MOFs under any desired conditions. Finally, we showcase a case study on exploring MOFs for flue gas separation using vacuum swing adsorption (VSA) under different temperatures.

### MPD-Based Adsorption Isotherm Database

The *NVT* + W approach as introduced previously involves the sampling of each macrostate, and its effectiveness has been demonstrated in several seminal studies recently reported in the literature.^52–56^ However, when it comes to building a large-scale adsorption database for a variety of adsorbates in thousands of adsorbents, sampling each possible macrostate is essentially impractical and computationally prohibitive. To reduce the computational cost, equal-space sampling where only macrostates separated at a fixed interval (e.g., Δ*N* = 4) are sampled as previously proposed by some of us^62^ may be applied. Δ*N* = 4 implies that only *N* = 0, 4, ..., *N*_max_ are being sampled, directly leading to a 4-fold less overall cost. For those macrostates that are not sampled, they could be estimated through interpolation of proximate macrostates, such as the cubic spline approach implemented in the scipy.interpolate function of the Python package as adopted herein. Using the adsorption of N_2_ in MOF ATIBIO as an example, Figure 1a demonstrates that equal-space samplings with Δ*N* = 4 yield isotherms that are in decent agreement with those from full MPD (Δ*N* = 1) and GCMC references, validating the effectiveness of the sampling strategy. However, as revealed in the logarithmic plot (Figure 1b), the accuracy of Δ*N* = 4 or even Δ*N* = 2 deteriorates at the low-pressure region, where deviations from full MPD or the GCMC reference are clearly visible. This discrepancy as shown in Figure S2 becomes even more prominent, as would be intuitively expected, when larger sampling intervals (i.e., Δ*N* = 8 and 16) are employed. Despite this limitation, opting for a greater Δ*N* is critically needed for developing a large-scale MPD-based database. Interestingly, it is found that the deviation primarily comes from the interpolation of the C values for macrostates with small *N*. As shown in Figure 1c,d, the interpolated C values from Widom deletion and insertion, respectively, at the reference condition are significantly deviated from the sampled data, particularly at *N* < 4. This behavior can be attributed to the steep change in C values for the low gas uptake at the reference pressure. As shown in Figure 1b, the adsorption uptake of N_2_ in ATIBIO is only ∼0.005 mol/kg (i.e., 0.0088 molecules/supercell) at the reference pressure of 0.1 bar, indicating that the structure strongly favors configurations with very few or no gas molecules present under such low-pressure conditions. As a result, when performing Widom deletion at *N* ≥ 1, nearly all Widom moves are likely to be accepted. Consequently, as reflected in the acceptance ratio for Widom deletion moves shown in Figure 1c, a sharp variation, jumping from zero to nearly the unity, occurs for macrostates between *N* = 0 and *N* = 1. While one may consider adapting a higher reference pressure to smooth out the sharp transition, this would then make Widom deletion moves for some structures, especially at low *N*, to be always rejected and thus make accurate statistics challenging. Therefore, there is no single ideal reference condition.

**Figure 1:**
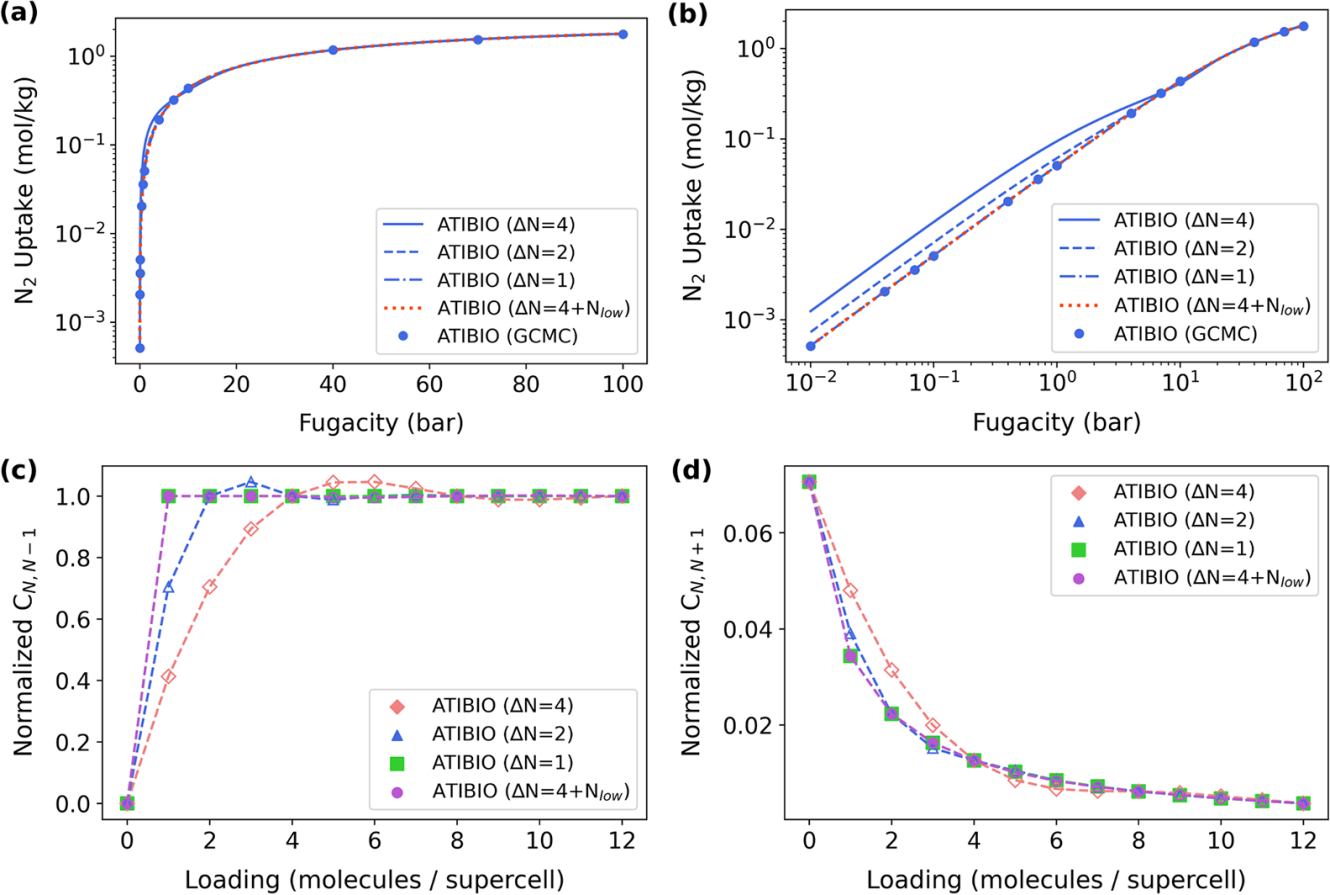
(a) Comparison between the MPD-based adsorption isotherms using different sampling schemes (e.g., Δ*N* = 4, 2, 1 (full MPD), or 4 + *N*_low_) and GCMC references in (a) semilog and (b) log–log scale for N_2_ in ATIBIO at 300 K. (c,d) C-matrix values normalized by the number of Widom moves (i.e., (c) normalized *C*_*N*, *N*–*1*_ and (d) normalized *C*_*N*, *N+*1_). Closed symbols represent values sampled from *NVT* + W calculations, while open ones refer to those determined from interpolation.

To this end, rather than aiming for a “best” reference condition, we choose a moderate reference pressure of 0.1 bar and 300 K, which is centered in our targeted temperature range and is relevant to carbon capture scenarios. Provided that the interpolation may not work well when there is a sharp change in the C value that is likely occurring in the low loading range, additional samplings on the near-empty macrostates (i.e., *N*_low_ = 1–3) are conducted. That is, under the equal-space sampling framework with Δ*N* = 4, macrostates of *N* = 1, 2, and 3 are also sampled. In addition, it is worth noting that the empty macrostate (*N* = 0) is always sampled explicitly. Although the acceptance ratios of Widom deletion moves at *N* = 0 are always zero, its Widom insertion statistics are crucial because *N* = 0 serves as the starting state for constructing the *C*_*N*, *N+1*_ curve in Figure 1d and for capturing the transition probability between *N* = 0 and 1. With this adjustment, Figure 1c-d evidently show that the resulting C values align very well with the full MPD data, demonstrating the effectiveness of this augmented approach as also shown in Figure 1a,b. This leads to an isotherm that perfectly resembles the GCMC-computed reference and is even more accurate than that of using Δ*N* = 2. It should be noted that the addition of near-empty macrostates only incurs minimal computational load, making the total simulation cost effectively equivalent to that of employing Δ*N* = 4.

With this approach, we have successfully developed an MPD-based adsorption database capable of offering accurate pure-component isotherms of CO_2_, N_2_, CH_4_, and CO at any desired temperature for ∼2900 MOFs. To further demonstrate the validity of the established MPD-based database with the strategic sampling approach using Δ*N* = 4 with *N*_low_ = 1–3, three MOF structures are selected for each adsorbate (CO_2_, N_2_, CH_4_, and CO) with their MPD-based isotherms compared to the GCMC-computed references. These 12 structures have a wide spectrum of Henry’s constants at 300 K, ranging from 2.82 × 10^–9^ to 4.30 × 10^–4^ mol/kg/Pa. As shown in Figure 2, the MPD-based uptakes at the reference temperature of 300 K are in excellent agreement with GCMC-computed data across full range of fugacities. Moreover, the MPD-based isotherms when reweighted to different temperatures (i.e., 280 and 330 K) also resemble, essentially perfectly, that of GCMC references. This highlights the robustness and versatility of our developed MPD-based database in accurately capturing adsorption behavior across diverse MOFs under a wide range of conditions. It is important to highlight that every GCMC data point as shown in Figure 2 requires separate simulations, and each calculation can take hours or even days to complete. For instance, to obtain one isotherm of CO_2_ (13 pressure points) in WEBREC at 300 K shown in Figure 2a, it takes nearly 500 h per CPU core. Conducting GCMC at different temperatures may therefore be impractical at database scale. By comparison, computing the MPD with our *NVT* + W workflow takes less than a hundred CPU-hours for the same structure, after which an isotherm under any desired temperatures can be generated in under a second with the same quality as the GCMC references shown in Figure 2. It should be noted that the methodology and database are readily generalizable to other porous materials and adsorbates. Though, it may remain challenging to extend the database to strongly associated adsorbates such as water. Although the *NVT* + W method has also been demonstrated to be a powerful approach to compute water adsorption in porous materials,^52–54,71^ the substantially longer sampling time may make the overall cost prohibitive at the present database scale.

**Figure 2:**
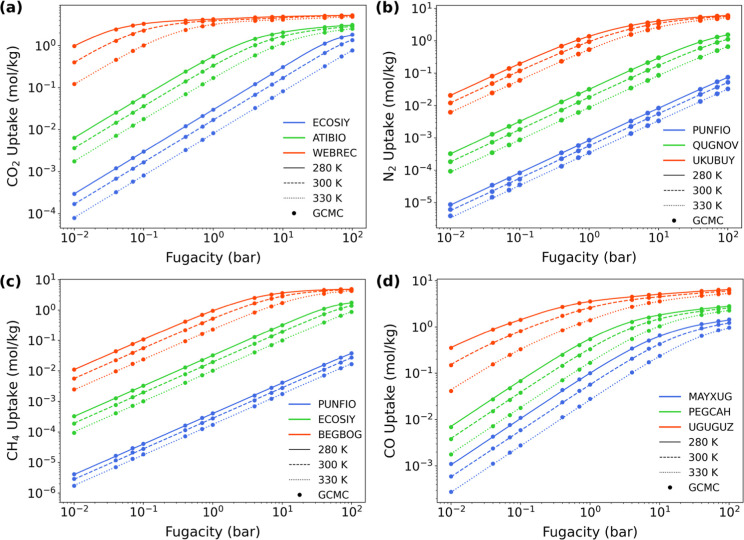
Comparison between MPD-based isotherms and GCMC-computed references for (a) CO_2_, (b) N_2_, (c) CH_4_, and (d) CO in selected MOFs at 280, 300, and 330 K.

### Mixture Isotherms

As separation processes inherently deal with mixtures, mixture isotherms are essential for assessing their performance metrics such as selectivity, working capacity, and regenerability to identify optimal adsorbents. With pure component adsorption isotherms, it has been a common practice to employ the ideal adsorbed solution theory (IAST) for predicting mixture isotherms. Although other computationally efficient models, such as extended Langmuir (EL)^72^ and dual-site Langmuir (DSL),^73^ could also be applied, the IAST has demonstrated strong predictive capability across diverse systems with a reasonable cost.^74–76^ Therefore, with the MPD-based pure-component isotherms at our disposal, we have further incorporated IAST to predict mixture isotherms. Distinctly, provided that the MPD-based database can offer uptakes at any desired pressures and temperatures, mixture adsorption isotherms can also be yielded at any targeted conditions. Moreover, MPD allows reweighting across a wide range of pressures with a theoretically unlimited number of data points at negligible computational cost. This enables a precise numerical integration of the spreading pressures needed in the IAST approach. Herein, the open-source pyIAST Python package^77^ is employed.

To probe the reliability of the resulting mixture isotherms from incorporating IAST with the MPD-based isotherms, using binary mixture adsorption of CO_2_/N_2_ as an example, we deliberately select four MOFs that exhibit distinct adsorption characteristics with their Henry’s constant ratios of CO_2_ over N_2_ varying from as small as ∼3 to as large as ∼100 at 300 K. Mixture isotherms for an equimolar CO_2_/N_2_ gas pair are computed using IAST with MPD-based pure-component isotherms at 280, 300, and 330 K, and compared against GCMC results. As shown in Figure 3a–f, the CO_2_ and N_2_ uptake values predicted using MPD-based IAST again closely align along the diagonal line across all conditions for all four MOFs, exhibiting excellent agreement with the GCMC references. The accuracy for CO_2_ demonstrated in Figure 3a,c,e is particularly noteworthy. This demonstrates that the MPD-based IAST framework can reliably capture binary adsorption behavior, offering a highly efficient alternative to repeated mixture GCMC simulations.

**Figure 3:**
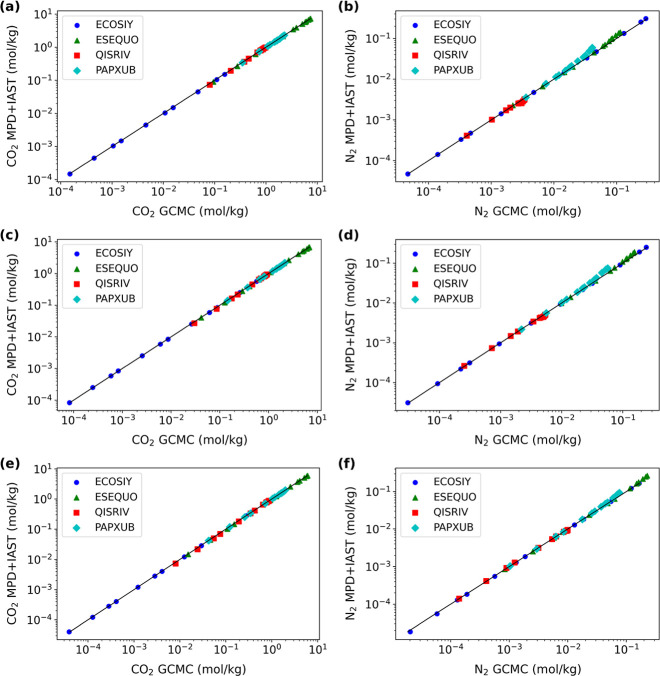
Comparison of uptake values for (a,c,e) CO_2_ and (b,d,f) N_2_ computed by IAST with MPD-based isotherms and direct GCMC simulations in four different MOFs at (a,b) 280 K, (c,d) 300 K, and (e,f) 330 K. The CO_2_/N_2_ mixture has a composition of 1:1.

Interestingly, noticeable, though slight, deviations may appear at higher pressures or lower temperatures for the weakly adsorbed N_2_. Take N_2_ in PAPXUB with a CO_2_/N_2_ Henry’s constant ratio of 62.8 at 280 K as an example, Figure 3b shows that the MPD-based IAST prediction begins to slightly overestimate N_2_ uptake compared to the GCMC reference at elevated pressures and lower temperatures. These discrepancies may be attributed to one of the key assumptions adopted in IAST: the framework offers the same accessible surface area to each adsorbate. At conditions where the adsorption capabilities of adsorbates differ significantly and when adsorption is nearly saturated (e.g., high pressure or low temperature), this assumption tends to break down since certain pores may be nearly exclusively occupied by the strongly adsorbed adsorbates, thereby blocking access for other gases.^78–80^ Nonetheless, despite this limitation, the integration of MPD-based isotherms with IAST still offers an efficient and reasonably accurate approach for predicting mixture adsorption under any desired conditions.

The above-noted limitation can in fact be alleviated by the application of two-dimensional *NVT* + W (2D *NVT* + W) as reported by some of us.^62^ Specifically, MPD for binary systems (i.e., 2D MPD) can also be obtained, which can be similarly reweighted to any desired pressure, temperature, and/or composition. As shown in Figure 4a, the CO_2_ and N_2_ uptake values from 2D MPD are in excellent agreement with GCMC references across the full fugacity range at 280 K. By contrast, the IAST fails to accurately predict the weakly adsorbed N_2_ where IAST tends to overestimate its uptake. Consequently, as further shown in Figure 4b, the computed CO_2_/N_2_ selectivities with IAST are underestimated whereas results from the 2D MPD resemble the GCMC references. More details of 2D NVT + W calculations and their results can be found in Supporting Information. However, the computational expense of 2D *NVT* + W, even with equal-space samplings, is simply prohibitive for constructing large-scale mixture adsorption isotherm databases. For instance, while sampling the needed macrostates for both pure components of CO_2_ and N_2_ in PAPXUB only takes approximately 170 h on a single CPU core, obtaining 2D MPD requires more than 4200 h. Therefore, integrating IAST with MPD for pure components evidently represents a more practical solution. 2D MPD can always be further obtained for certain MOFs of interest at a later stage.

**Figure 4:**
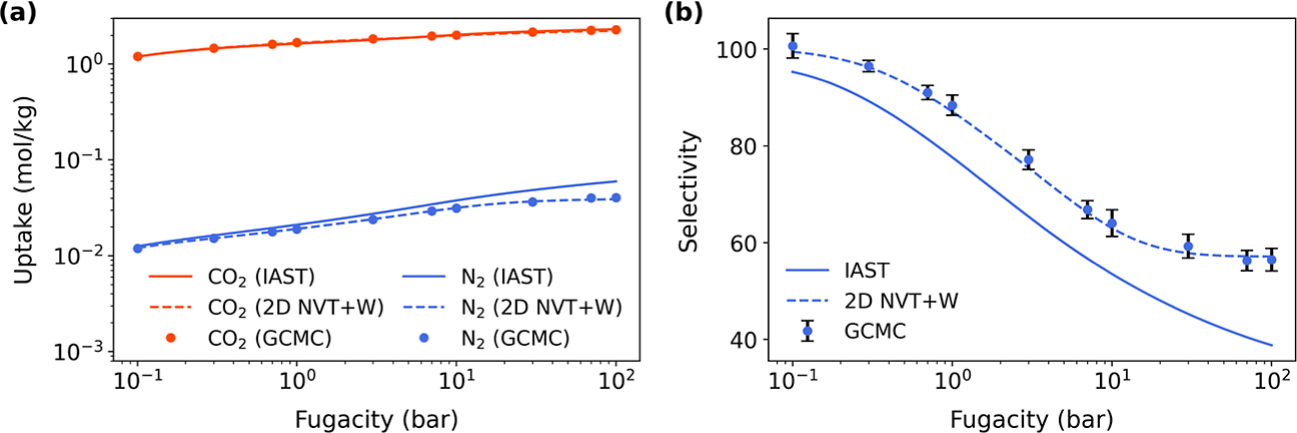
(a) Adsorption isotherms of CO_2_/N_2_ (1:1) mixtures and (b) the corresponding selectivity of CO_2_ over N_2_ determined from MPD-based IAST and 2D NVT + W at 280 K in PAPXUB. GCMC-determined references are also shown for comparison.

In addition to binary systems, we have also employed IAST in conjunction with MPD-based pure-component isotherms to generate ternary mixture adsorption isotherms. As shown in Figure S3, two structures are selected for validation of ternary IAST. Similar to the binary cases, CO_2_ uptakes are consistently well captured across various conditions. For more weakly adsorbed components like N_2_ and CH_4_, the IAST predictions may also deviate slightly at high pressures or low temperatures, again due to its assumption of equal adsorbate accessibility within the pore space. Nevertheless, the overall agreement with ternary GCMC simulations remains strong, validating the MPD + IAST integration as a fast and reliable approach for multicomponent isotherms.

### Online MPD-Based Adsorption Database Web Site

An interactive Web site^81^ has also been established along with this study. As shown in Figure 5a, the Web site homepage allows users to interactively explore MOFs based on structural properties such as pore limiting diameter (PLD), largest cavity diameter (LCD), density, accessible surface area (both gravimetric and volumetric), and void fraction. A filtering panel with adjustable sliders enables efficient narrowing of materials according to user-targeted criteria. Upon selecting a MOF, its individual webpage also offers a 3D crystal structure visualization along with a detailed summary of structural characteristics. Moreover, as shown in Figure 5b–d, extensive adsorption data including single-component, binary, and ternary isotherms are made available. For pure-component adsorption isotherms, Figure 5b shows that both linear and logarithmic scale graphs over a fugacity range of 0.00001–100 bar and temperatures from 280 to 340 K are displayed. As for binary and ternary gas mixtures shown in Figure 5c,d, respectively, the database offers IAST-computed mixture isotherms per MPD-based pure component isotherms with user-defined adsorbate combinations and molar ratios. To facilitate further analyses, users can also download tabulated isotherm data by clicking the “Download isotherm” button available beneath each graph.

**Figure 5:**
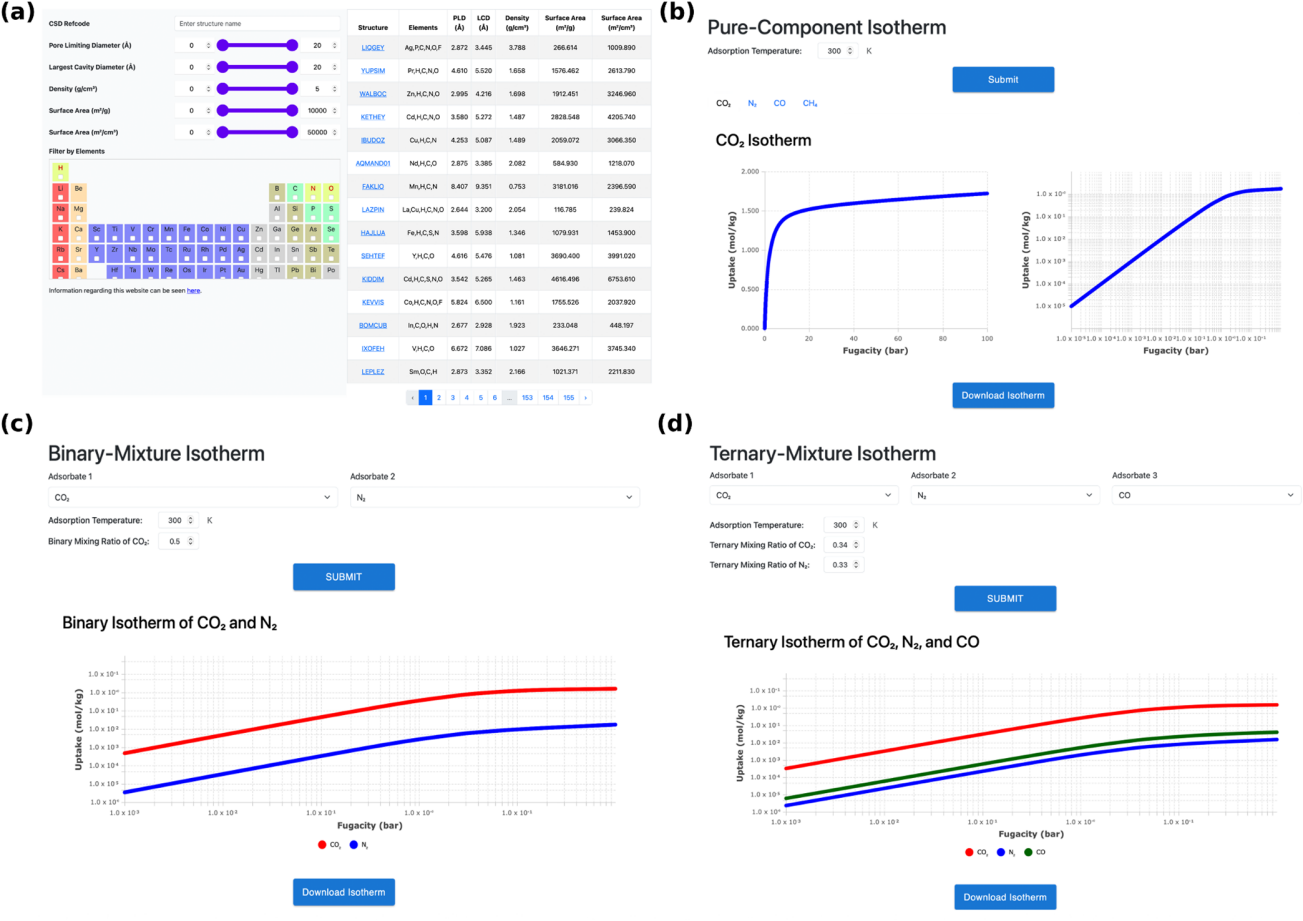
Screenshots of (a) the homepage with structural properties of each MOF and search criteria as well as (b–d) adsorption isotherms for (b) pure component, (c) binary mixture, and (d) ternary mixture for CO_2_, N_2_, CH_4_, and CO at any user-defined conditions and compositions.

### Case Study–Flue Gas Separation

To demonstrate the utility of the established MPD-based adsorption database, a case study on flue gas separation is carried out. Herein, we consider a flue gas molar composition of CO_2_/N_2_ = 15/85 to be separated through a VSA process where the adsorption and desorption pressures are assumed to be at 1 and 0.1 bar, respectively. In this illustrative case, the operating pressures are fixed as we do not attempt to optimize the operating conditions. Instead, we explore the VSA process under varied temperatures over a range of 280–350 K. This case study highlights a key advantage of our database—it enables rapid evaluation of separation performance and structure-performance relationship across temperatures without rerunning molecular simulations. Traditional models like Langmuir may allow extrapolation, but may fall short for varying temperatures or complex mixtures. In contrast, our MPD-based framework provides direct access to multicondition and high-fidelity data.

To evaluate the effectiveness of the separation process, several key performance metrics, including working capacity (Δ*N*), selectivity ( $S_{ads,{CO}_{2}/N_{2}}$ ), adsorbent performance score (APS), and regenerability (*R*%), as defined below in eqs 7–10, respectively, are calculated.

7 $$\Delta N=N_{ads}-N_{des}$$

8 $$S_{ads,{CO}_{2}/N_{2}}=\frac{N_{{CO}_{2}}}{N_{N_{2}}}/\frac{y_{{CO}_{2}}}{y_{N_{2}}}$$

9 $$APS{=\;S}_{ads,{CO}_{2}/N_{2}}\times \Delta N_{{CO}_{2}}$$

10 $$R\%=\frac{\Delta N_{{CO}_{2}}}{N_{ads,{CO}_{2}}}\times 100\%$$

In these equations, *N*_ads_ and *N*_des_ represent the gas uptakes at the adsorption and desorption pressure, respectively, and *y* denotes the composition of species in the gas mixture.

Figure 6a presents the relationship between selectivity and working capacity across a temperature range of 280–350 K. As shown, several MOFs exhibit high selectivity, ranging from 10^4^ to 10^6^ at each temperature. Notably, approximately 300 MOFs at 280 K show selectivity values exceeding 1,000, and about 110 MOFs still surpass this value at 350 K. However, these highly selective materials often suffer from limited working capacities, which may diminish their practical applicability. This may arise from their too strong affinity toward CO_2_, which hampers desorption under low-pressure conditions. In terms of working capacity, Figure 6a displays that lower temperatures generally lead to higher values. This trend can be explained since as temperature decreases, the adsorption of gases becomes more favorable due to the exothermic nature of the process, leading to higher gas uptakes at the adsorption pressure. While these observations highlight how different separation matrics dominate under different conditions, evaluating adsorbents based on a single criterion can be misleading. Therefore, to better capture overall separation performance, we also evaluate regenerability (*R*%), which is a critical consideration for cyclic adsorption processes,^82^ as well as employ the APS metric defined as the product of selectivity and working capacity. As shown in Figure 6b, the percentage of MOFs with *R*% > 85% varies significantly with temperature. At low temperatures (280–290 K), only ∼30% of MOFs achieve *R*% > 85%, indicating relatively poor regeneration potential. This phenomenon similarly arises from the stronger adsorption at low temperature. Although CO_2_ adsorption is enhanced, desorption becomes more difficult during the regeneration step at 0.1 bar, resulting in lower *R*%. In contrast, over 65% of MOFs meet this threshold at higher temperatures (340–350 K). However, this improvement in regenerability comes at the cost of performance, as the average APS drops from 1732.10 mol/kg at 280 K to 801.40 mol/kg at 350 K, reflecting a decline in overall separation efficiency under warmer conditions. Overall, these results suggest a trade-off: low-temperature VSA offers higher working capacity and APS but suffers from limited regenerability.

**Figure 6:**
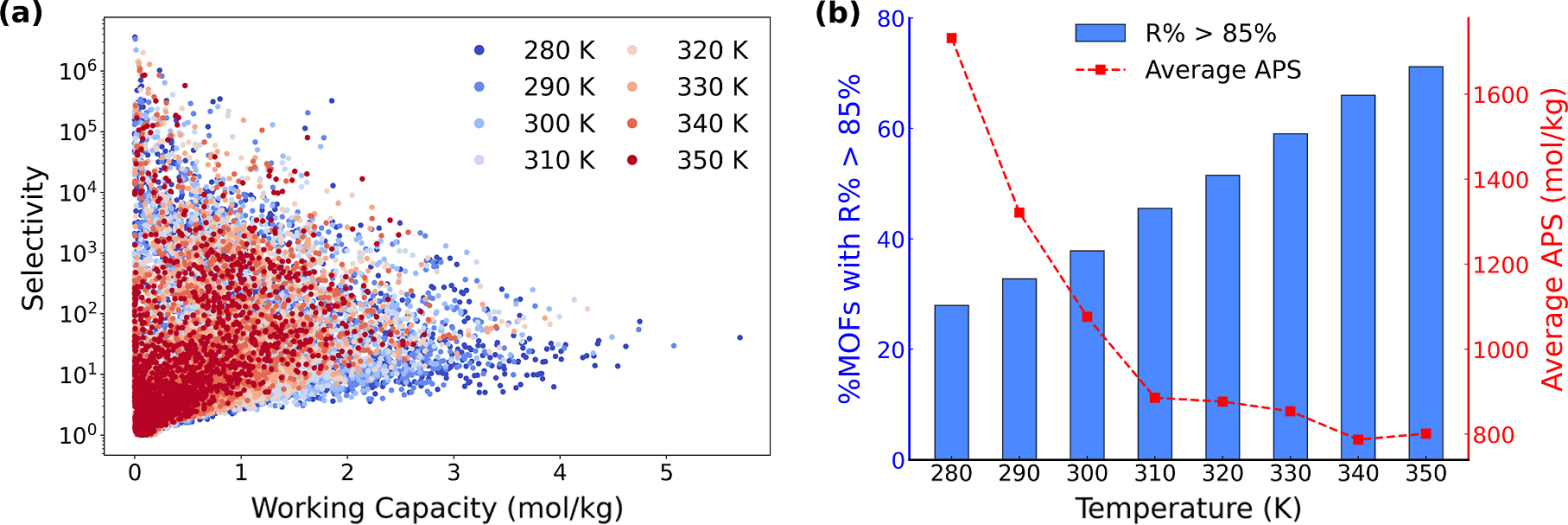
(a) Selectivity vs working capacity. (b) Percentage of MOFs with regenerability (*R*%) greater than 85% and average adsorbent performance score (APS) of studied MOFs for CO_2_/N_2_ = 15/85 mixture under VSA condition computed at an adsorption (desorption) pressure of 1 (0.1) bar from 280 to 350 K.

To further examine some structural properties (e.g., largest cavity diameter (LCD), pore limiting diameter (PLD), void fraction, and accessible surface area (ASA)) of the top-performing MOFs at each temperature, as shown in Figure 6, the average values of these properties are plotted for MOFs with either the top 10% working capacity (Δ*N*) or regenerability *R*% > 85% across the temperature range of 280–350 K. Generally, Figure 7 reveals a clear trend that at lower temperatures, top-performing MOFs tend to possess larger pores, higher void fractions, and greater accessible surface areas. Specifically, MOFs that exhibit high working capacity consistently show more porous structures at 280 to 330 K. Since adsorption is stronger at lower temperatures as noted above, larger pores and greater surface area enable more CO_2_ to be adsorbed at the adsorption pressure, thereby boosting CO_2_ working capacity. On the other hand, from 330 to 350 K, the variation of structural properties with temperature becomes less prominent. Figure S4 also indicates that at lower temperatures, the median working capacity increases significantly from 0.78 (280 K) to 1.99 mol/kg (280 K) as LCD increases from 4.0–4.5 Å to 6.0–6.5 Å. In contrast, at higher temperatures, Δ*N* varies only slightly across different LCD intervals, suggesting that surface chemistry, rather than pore geometry, may play a more dominant role in governing adsorption performance. Additionally, the structure-performance relationship for MOFs with high adsorbent performance score (APS) is also analyzed with results shown in Figure S5. Based on the results, the structural properties for the MOFs of top 10% APS show little correlation to temperatures compared to those top-Δ*N* structures. Since APS is the product of selectivity and working capacity, the APS values might be dominated by some extremely high-selectivity MOFs as shown in Figure 6a. To obtain high selectivity, strong confinement effect is necessary regardless of the temperature to enhance the adsorption of CO_2_. Hence, in Figure S5, top-APS structures have relatively small pore size, void fraction and ASA at every temperature.

**Figure 7:**
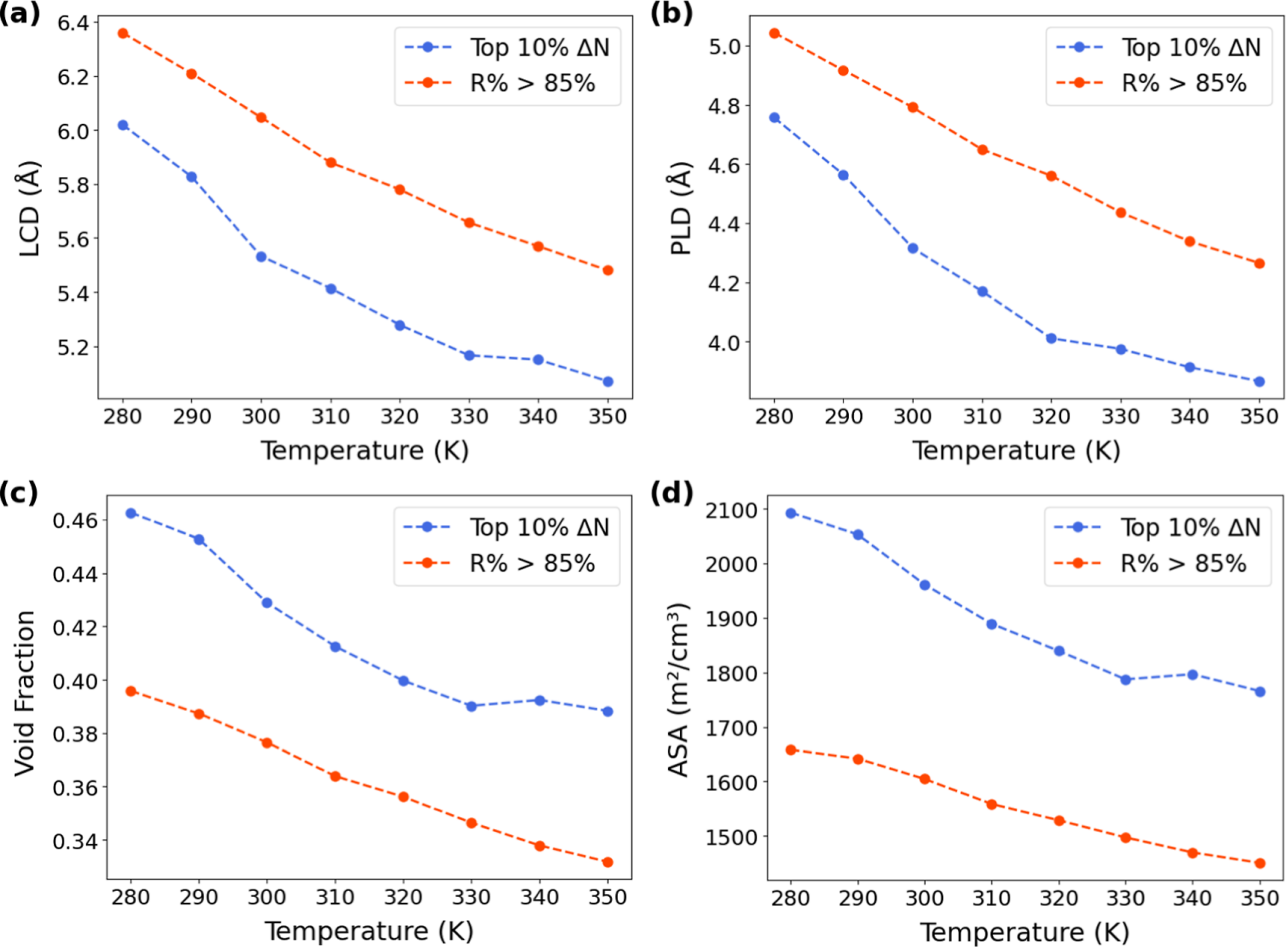
Average (a) LCD (b) PLD (c) void fraction (d) ASA for MOFs of top 10% working capacity or *R*% > 85% at temperatures ranging from 280 to 350 K.

A similar trend is observed for MOFs with high regenerability as also shown in Figure 7, especially at lower temperatures. Structures that can achieve *R*% > 85% under these conditions also tend to have more open frameworks. This appears reasonable given that larger pore systems reduce the strength of confinement and allow for easier desorption. In contrast, in tighter pores, the strong CO_2_–framework interactions at low temperatures can hinder desorption at the desorption pressure. Interestingly, MOFs in the top 10% working capacity are found to have larger LCD and PLD values compared to those with *R*% > 85% (Figure 7a,b), while void fraction and ASA show opposite trends (Figure 7c,d). This indicates that for those MOFs of higher working capacities, the density of favorable pores (though smaller in size) is higher, leading to greater void fraction as well as ASA.

Overall, as temperature rises, both top-Δ*N* and high-*R*% subsets shift toward smaller average structural dimensions, consistent with the general decrease in adsorption strength and total uptake. Therefore, MOFs with larger and more accessible pore structures are better suited for high working capacity and effective regeneration at low temperatures.

Beyond enabling rapid identification of promising MOFs and uncovering structure-performance relationships, it is important to point out that this MPD-based adsorption database may be especially valuable for adsorption process optimizations. For instance, in a recent study on the process optimization for ternary natural gas separation, Yoon et al.^83^ pointed out that during a full process cycle, the gas composition, temperature, and pressure can vary significantly across different steps and may deviate substantially from the original feed conditions. This variability presents a major challenge, as accurate mixture data are needed at each of these varying conditions. Our MPD-based framework is well poised to address this difficulty by providing direct access to mixture isotherms across a continuous range of conditions, including varied temperatures, pressures, and gas compositions. This enables efficient, condition-specific optimization without having to carry out new simulations for each process state, thereby streamlining the consideration of both materials’ properties and realistic adsorption processes.

## Conclusions

This study establishes for the first time a versatile large adsorption database for four key small molecules in thousands of MOFs. Unlike traditional GCMC approaches that require repeated simulations for each condition, our method leverages the macrostate probability distribution (MPD), which only needs to be generated once at a reference temperature and fugacity. Once computed, the MPD can be reweighted analytically and instantly to yield accurate adsorption isotherms across a broad range of temperatures and pressures. In this study, to ensure computational efficiency for obtaining MPD, an effective equal-space sampling strategy (Δ*N* = 4) is employed. Enhanced samplings are also applied by explicitly including near-empty macrostates (*N*_low_ = 1–3). This approach strikes a balance between accuracy and cost, and achieves excellent agreement with GCMC-computed references. To extend the database to gas mixtures, we also integrate the pure-component MPD data with ideal adsorbed solution theory (IAST), enabling rapid and reasonably accurate predictions of mixture isotherms. While minor deviations may arise for weakly adsorbed species particularly at high pressures or low temperatures, IAST remains effective for large-scale screening across diverse materials. Moreover, we develop a user-friendly and powerful online Web site^81^ that provides access to structural properties and pure-component (CO_2_, N_2_, CH_4_, and CO) as well as their mixture isotherms across a wide range of conditions or compositions for over 2900 MOFs. The study also showcases the utility of the MPD-based database through a case study on flue gas separation. Key separation performance metrics, including working capacity, selectivity, APS, and regenerability, through VSA process under a wide range of temperatures can be rapidly evaluated. This capability not only accelerates the identification of promising MOFs, but also allows for systematic investigation of structure-performance relationships as a function of temperature, offering valuable insights into how structural features influence adsorption behavior under different operating conditions. Overall, the MPD-based database offers an efficient and scalable platform for instantly acquiring adsorption isotherms under any desired conditions, enabling high-throughput screening and systematic analysis of structure-performance relationships for adsorption-related applications. This may be particularly powerful for process optimization tasks that require adsorption data under dynamically changing conditions. Looking ahead, this database can be straightforwardly expanded to include a wider variety of adsorbates (e.g., longer alkane) and porous materials (e.g., the newer CoRE MOF 2024 database^24^ that includes more MOFs), broadening its utility for facilitating the development of adsorption technology.

## Supplementary Material



## Notes

Additional tables and figures referred in the main text, including force field parameters, comparison of two adopted cutoff distance settings, comparison of different equal-space sampling schemes, ternary mixture isotherms obtained from MPD-based IAST, analysis of working capacity at different LCD values, and the structure-performance relationship for top-APS MOFs. Details of the 2D *NVT* + W calculations are also included (PDF)
