# Supplementary material for: Queryable Gaseous Adsorption Properties of Pure Components and Mixtures in Metal–Organic Frameworks
Source: Langmuir. 2025 Nov 22;41(48):32285–97. doi: 10.1021/acs.langmuir.5c03717 (PMC12874540; doi:10.1021/acs.langmuir.5c03717)
Supplement: Supplementary file 1 [file la5c03717_si_001.pdf]

Supporting Information

for

Queryable Gaseous Adsorption Properties of Pure Components and Mixtures in Metal-Organic Frameworks

Jui Tu[a], Chi-Chun Tang[a], Pi-Chien Chuang[a], and Li-Chiang Lin,[a,b,]*

[a]Department of Chemical Engineering, National Taiwan University, No. 1, Sec. 4, Roosevelt Road, Taipei 106319, Taiwan

[b]William G. Lowrie Department of Chemical and Biomolecular Engineering, The Ohio State University, Columbus, Ohio 43210, United States

[*]Email: lclin@ntu.edu.tw

Table of contents

1. Tables referred in the main text S3

2. Figures referred in the main text S4

3. 2D NVT+W S7

1. Tables referred in the main text

Table S1. Parameters of the adopted TraPPE[1] CO_2 model.

Site	ε/k_B (K)	σ (Å)	charge (e^-)	x (Å)	y (Å)	z (Å)
O	79.0	3.05	-0.3253	0.0	0.0	1.16
C	27.0	2.80	0.6506	0.0	0.0	0.0
O	79.0	3.05	-0.3253	0.0	0.0	-1.16

Table S2. Parameters of the adopted TraPPE[1] N_2 model.

Site	ε/k_B (K)	σ (Å)	charge (e^-)	x (Å)	y (Å)	z (Å)
N	36.0	3.31	-0.4050	0.0	0.0	0.55
N_com	0	0	0.8100	0.0	0.0	0.0
N	36.0	3.31	-0.4050	0.0	0.0	-0.55

Table S3. Parameters of the adopted TraPPE-UA[2] CH_4 model.

Site	ε/k_B (K)	σ (Å)	charge (e^-)	x (Å)	y (Å)	z (Å)
CH_4	148.0	3.73	0	-	-	-

Table S4. Parameters of the adopted ESM-MM[3] CO model. "X" represents massless pseudo-site.

Site	ε/k_B (K)	σ (Å)	charge (e^-)	x (Å)	y (Å)	z (Å)
O	40.5	3.08	-0.209	0.0	0.0	1.129
C	40.2	3.52	1.040	0.0	0.0	0.0
X	0.0	0.0	-0.831	0.0	0.0	-0.336

2. Figures referred in the main text

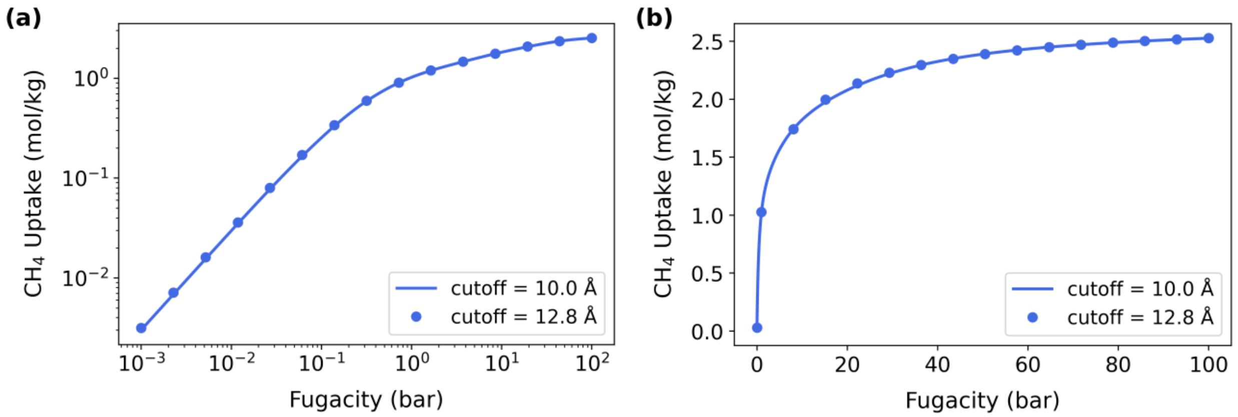

Figure S1. Comparison between NVT+W-computed adsorption isotherms adopting cutoff distance of 10.0 and 12.8 Å with and without tail correction, respectively, in (a) log-log and (b) normal scale for CH_4 in LEWVAL at 300 K.

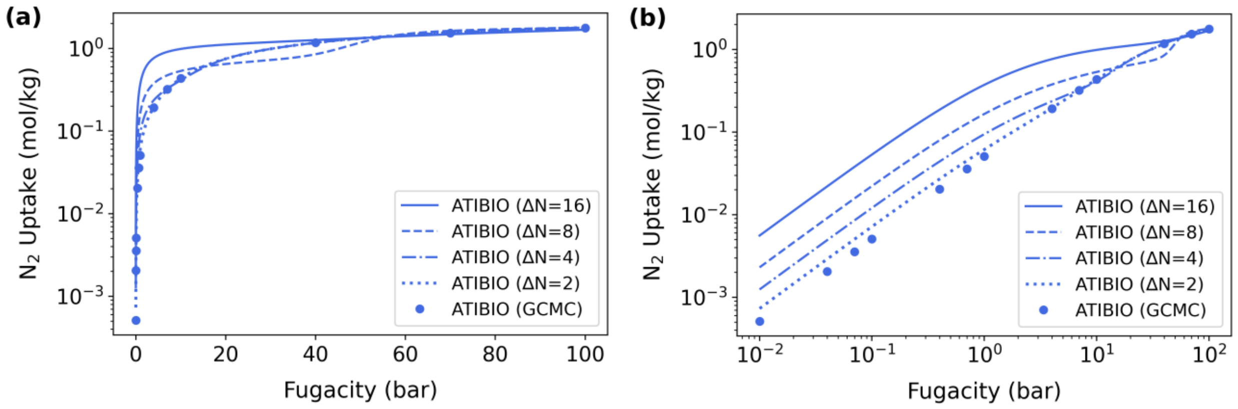

Figure S2. Comparison between the NVT+W-computed adsorption isotherms using different sampling schemes (e.g., $\Delta N = 2, 4, 8, \text{or } 16$) and GCMC references in (a) semi-log and (b) log-log scale for N_2 in ATIBIO at 300 K.

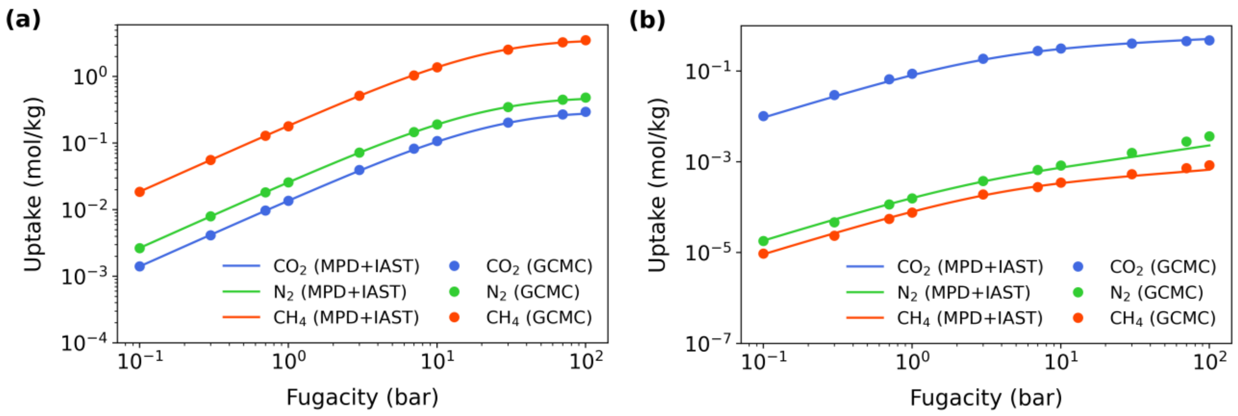

Figure S3. Adsorption isotherms of $CO_2/N_2/CH_4$ (34:33:33) mixtures determined from MPD-based IAST at 300 K in (a) BEGBOG and (b) PUNFIO. GCMC-determined references are also shown for comparison.

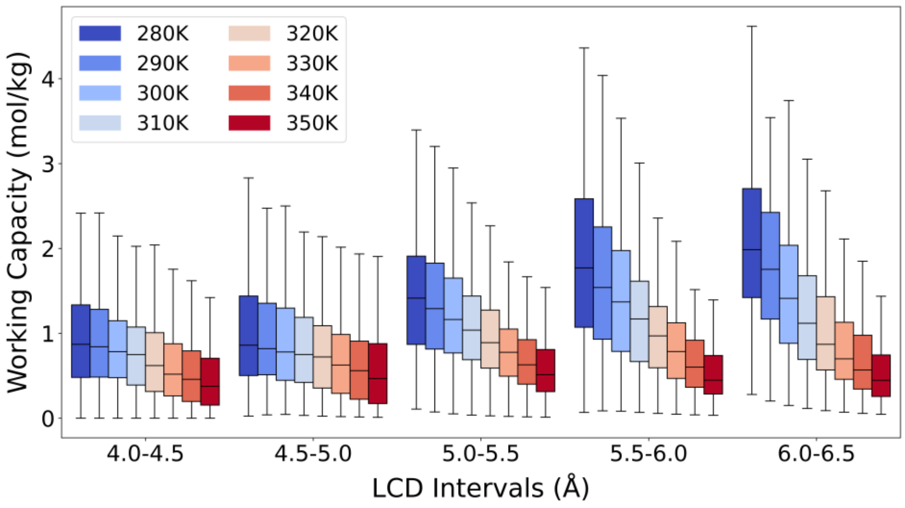

Figure S4. Working capacity of MOFs within different LCD intervals for separating CO_2/N_2 (15/85) mixture through VSA with an adsorption (desorption) pressure to be 1 (0.1) bar at varying temperatures ranging from 280 to 350 K.

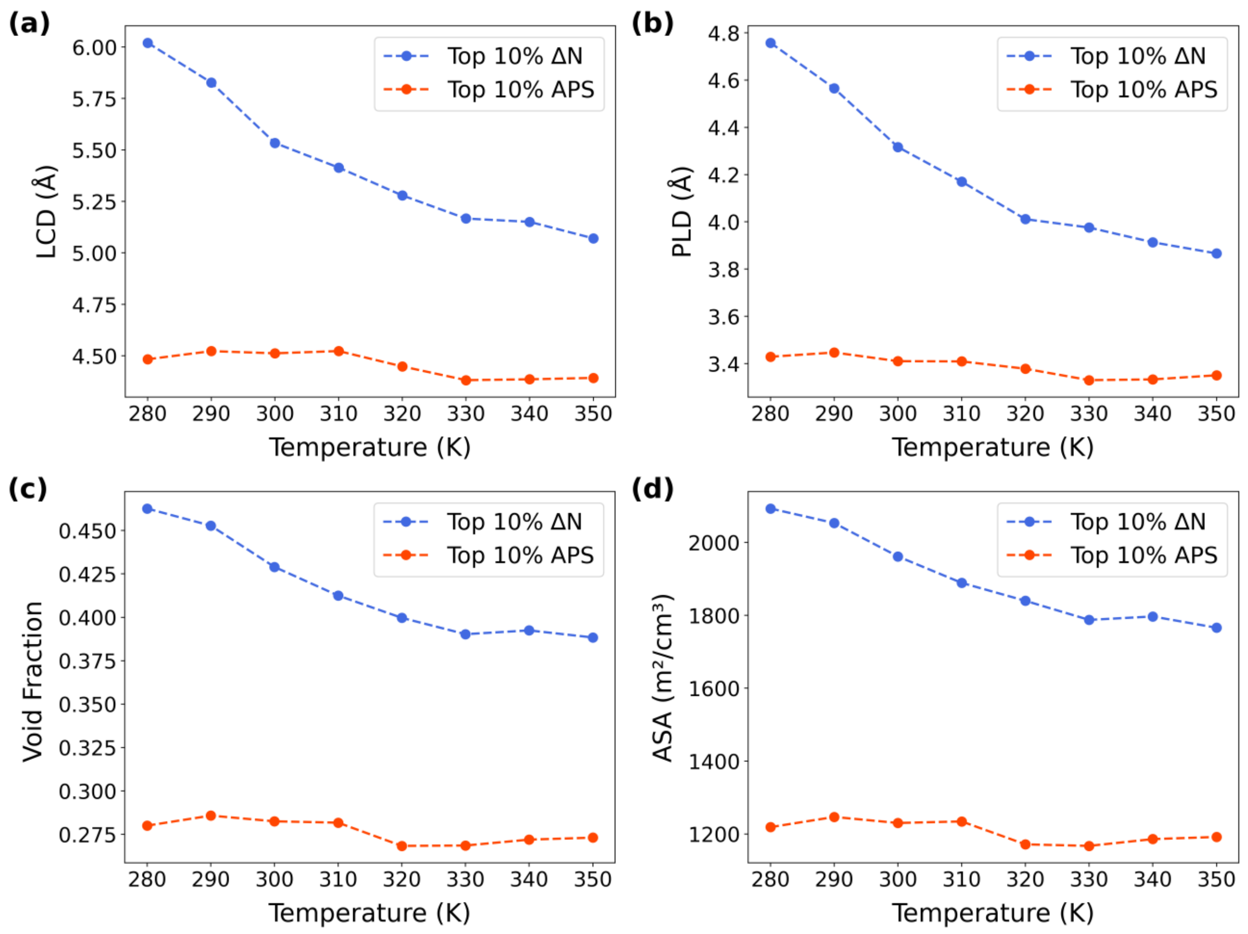

Figure S5. Average (a) LCD (b) PLD (c) void fraction (d) ASA of top 10% MOFs in terms of working capacity or adsorbent performance score (APS) at temperatures ranging from 280 K to 350 K.

3. 2D NVT+W

For 2D NVT+W simulations, the reference condition is selected to be 300 K and a total pressure of 0.2 bar with 1:1 molar ratio. Similar to one-dimensional NVT+W, the N_{max} for each adsorbate is set to be the corresponding saturation loading N_{sat} determined by GCMC simulations under a fugacity value of 100 bar and at a temperature of 275 K. The macrostates within the triangular region defined by vertices $(0,0)$, $(N_1^{max}, 0)$, and $(0, N_2^{max})$ where N_1^{max} and N_2^{max} represent the N_{max} for adsorbate 1 and 2 respectively, are sampled to compute 2D MPD. The 2D MPD can then be reweighted to any desired pressure, temperature, or molar ratio. The readers are referred to our previous study[4] for more details regarding the theoretical details of the approach. In addition to the example shown in the main text, **Figure S6** further shows the adsorption isotherms of racemic CO_2/N_2 mixtures predicted by the 2D NVT+W method and IAST for two more selected MOFs – ANENIR and QUGNOV. Both of these MOFs have a notably stronger adsorption of CO_2 over N_2. **Figure S6a** again shows that IAST underpredicts the N_2 uptake across the full pressure range, resulting in an overestimated CO_2 selectivity. In contrast, the 2D NVT+W simulation accurately computes the mixture gas uptakes of both CO_2 and N_2. Similarly, **Figure S6b** also shows that IAST fails to capture the more weakly adsorbed N_2 at higher pressures. The 2D NVT+W method again successfully generates accurate mixture isotherms. These comparisons highlight the limitations of IAST, specifically in systems when there exists a large difference in the adsorption strength of two adsorbates. Despite so, as noted in the main text, it is computationally prohibited to obtain 2D MPD for each pair of adsorbates in every MOF. The utilization of IAST per MPD-based pure component isotherms still represents a much more effective approach to determine mixture adsorption properties.

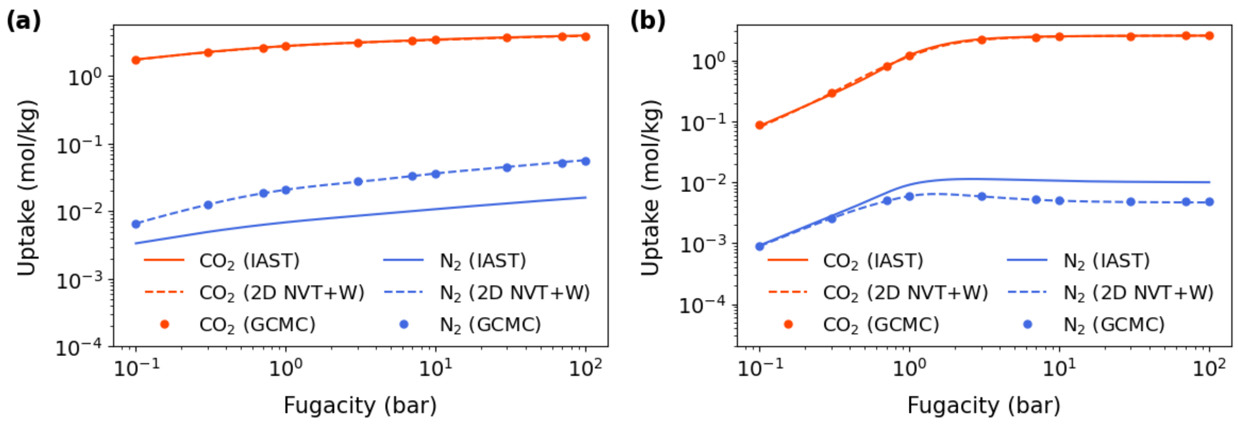

Figure S6. Comparison of the adsorption CO_2/N_2 (1:1) mixture isotherms obtained via MPD-based IAST and 2D NVT+W in (a) ANENIR and (b) QUGNOV.

4. References

1. Potoff, J. J.; Siepmann, J. I. Vapor–liquid equilibria of mixtures containing alkanes, carbon dioxide, and nitrogen. *AIChE J.* **2001**, *47* (7), 1676–1682. DOI: 10.1002/aic.690470719.
2. Martin, M. G.; Siepmann, J. I. Transferable potentials for phase equilibria. 1. United-atom description of n-Alkanes. *J. Phys. Chem. B* **1998**, *102* (14), 2569–2577. DOI: 10.1021/jp972543+.
3. Cho, E. H.; Lin, L.-C. Electrostatic potential optimized molecular models for molecular simulations: CO, CO_2, COS, H_2S, N_2, N_2O, and SO_2. *J. Chem. Theory Comput.* **2019**, *15* (11), 6323–6332. DOI: 10.1021/acs.jctc.9b00653.
4. Chen, H.-C.; Lin, L.-C. Computing mixture adsorption in porous materials through flat histogram Monte Carlo methods. *Langmuir* **2023**, *39* (43), 15380–15390. DOI: 10.1021/acs.langmuir.3c02466.
